# Bone mineral density after treatment for gastric cancer: Endoscopic treatment versus gastrectomy

**DOI:** 10.1097/MD.0000000000009582

**Published:** 2018-01-05

**Authors:** Hye-Mi Noh, Jun-Hyun Yoo, Ji Young Jeong, Yong Soon Park

**Affiliations:** a Department of Family Medicine, Hallym University Sacred Heart Hospital, Hallym University College of Medicine, Anyang; b Department of Family Medicine, Samsung Medical Center, Sungkyunkwan University School of Medicine, Seoul; c Department of Family Medicine, Chuncheon Sacred Heart Hospital, Hallym University College of Medicine, Chuncheon, Republic of Korea.

**Keywords:** bone mineral density, endoscopic gastrointestinal surgery, gastrectomy, gastric cancer, survivors

## Abstract

Changes in bone metabolism among gastric cancer survivors have long been recognized. The aim of our study was to clarify the changes of bone mineral density (BMD) among gastric cancer survivors who underwent endoscopic resection or gastrectomy. Forty-nine patients diagnosed with tumor, node, and metastasis (TNM) stage 1 gastric cancer with pathologic confirmation, who underwent BMD measurement just before the procedure, and had no prior osteoporosis treatment, were studied. BMD was measured with dual energy x-ray absorptiometry before and after treatment. Laboratory tests were performed using fresh serum, and serum levels of alkaline phosphatase, albumin, calcium, and phosphorus were measured. We used a nested case-control design to compare groups. Of the 49 patients, 34 underwent gastrectomy and 15 underwent endoscopic treatment. There were no differences in baseline clinical characteristics, including BMD, and biochemical data between groups. The mean and median follow-up intervals for BMD measurement were 32.6 months (standard deviation, 16.5) and 31.0 months (interquartile range: 21.5, 41.0), respectively. The follow-up BMDs of the femoral neck and total hip were lower in the gastrectomy group (*P* = .010 and .011, respectively). The percentage changes in BMD for the lumbar spine, femoral neck, and total hip were −3.30%, −1.52%, and 0.40%, respectively, in the endoscopic treatment group, and −7.17%, −6.30%, and −3.49%, respectively, in the gastrectomy group. Bone loss of the lumbar spine and femoral neck were greater in the gastrectomy group (*P* = .028 and .022, respectively). BMD is lower after gastrectomy than after endoscopic treatment among early stage gastric cancer survivors.

## 1 Introduction

Gastric cancer survivors have many risk factors for osteoporosis, such as malnutrition, weight loss, and chemotherapy. Osteoporosis is an important health problem after gastrectomy, which could result in complications, including pain, compression, and fracture. The prevalence of osteoporosis has been reported to be higher in gastric cancer survivors than in the general population.^[1]^ Several studies have reported that bone mineral density (BMD) was lower in patients who underwent gastrectomy than in age- and sex-matched healthy controls.^[2,3]^ However, a longitudinal study conducted in Sweden found no differences in BMD between patients who underwent gastrectomy and a reference population at 5 and 8 years after gastrectomy.^[4]^ In contrast, a prospective study of Korean gastric cancer survivors demonstrated that BMD was significantly lower 12 months after gastrectomy than before gastrectomy.^[5]^ However, these studies had limitations. The follow-up intervals were relatively short and confounding factors of bone loss, such as age, cancer burden (tumor stage), and the cytotoxic effects of chemotherapy, could not be adjusted owing to small sample sizes.

Endoscopic resection is becoming the primary procedure for the treatment of early gastric cancer in cases that can be treated with this procedure.^[6]^ Considering that endoscopic resection could preserve the function of the stomach, this procedure might prevent the complications that result from gastrectomy. However, a few studies have compared the health problems of gastric cancer survivors between endoscopic treatment and gastrectomy.

The current study aimed to clarify the changes in BMD among gastric cancer survivors who underwent endoscopic treatment and those who underwent gastrectomy in order to determine which procedure is more appropriate to maintain bone health.

## 2 Methods

### 2.1 Study subjects and design

The study included 49 patients who were diagnosed with tumor, node and metastasis (TNM) stage 1 gastric cancer with pathologic confirmation and who underwent BMD measurement just before gastrectomy or endoscopic treatment at the Health Promotion Center of Samsung Medical Center in Seoul, Korea between January 1, 2002 and December 31, 2007. The inclusion criteria were diagnosis of gastric adenocarcinoma on esophagogastroduodenoscopy with pathologic confirmation; performance of curative gastrectomy (total gastrectomy and subtotal gastrectomy) or endoscopic treatment (endoscopic mucosal resection and endoscopic submucosal dissection) and follow-up without recurrence; without treatment for osteoporosis and history of fracture before the diagnosis of gastric cancer; no diseases, such as renal failure, thyroid disease, liver cirrhotic disease, hormone replacement therapy, and immobility, which can affect BMD; measurement of baseline BMD before surgical or endoscopic treatment and follow-up BMD after treatment. To minimize the influences of cancer burden or chemotherapy on bone loss, we selected early stage gastric cancer survivors as the study population. We used the nested case-control design to compare the gastrectomy and endoscopic treatment groups. The study subjects were divided to 2 treatment groups according to whether the subject meets the standard indication criteria for endoscopic treatment.^[7]^ The standard criteria for selection of subjects who are appropriate for the endoscopic treatment are below: well or moderately differentiated adenocarcinoma; confined to the mucosa; smaller than 2 cm for superficially elevated type lesions; smaller than 1 cm for the flat and depressed type lesions; without ulcer or ulcer scar; and without venous or lymphatic involvement. The study was reviewed and approved by the Samsung Medical Center Institutional Review Board (2011-08-073).

### 2.2 Measurements

BMDs (g/cm^2^) of the lumbar spine (L1–L4), left femoral neck, and total hip were measured with dual energy x-ray absorptiometry (DXA; Delphi W, Hologic Inc., Bedford, MA). All measurements were recorded by a well-trained technician. The coefficients of variation for the BMD measurements were ≤1.0% for the lumbar spine and hip.

Laboratory tests were performed using fresh serum. Serum levels of alkaline phosphatase, albumin, calcium, and phosphorus were measured using a Hitachi-7600 system (Hitachi, Tokyo, Japan). Serum calcium was corrected for changes in serum albumin concentration according to the following formula: corrected calcium (mg/dL) = actual calcium + (4 − serum albumin) × 0.2.

Body weight (kg) and height (cm) were measured to the nearest 0.1 kg and 0.1 cm, respectively, with the patients in light clothing and without shoes. Body mass index (BMI) was calculated as weight divided by height squared (kg/m^2^). Anthropometric measurements were performed using the bioelectrical impedance analysis method (InBody, Biospace, Seoul, Korea).

A self-administered questionnaire was used to obtain baseline characteristics such as history of medical treatment or surgery, current medication (calcium supplements, anti-resorptive medication, or hormone replacement therapy), menopausal status, and health-related behaviors (smoking and alcohol consumption). Based on the smoking history, we classified the patients into current, past, and never smokers. Furthermore, based on the weekly frequency of alcohol consumption, we classified the patients into ever drinkers (≥1 time/week) and never drinkers (<1 time/week).

### 2.3 Statistical analysis

Continuous data are presented as means and standard deviations (SDs), and categorical data are presented as frequencies and proportions, as appropriate. Continuous variables were assessed for normality using graphical tools and the Shapiro-Wilk test. Comparisons between the gastrectomy and endoscopic treatment groups were performed by using the *t* test for continuous data and the *χ*^*2*^ test with Fisher exact test for categorical data. As an exception, BMD intervals were expressed as medians and interquartile ranges, and compared using the Mann-Whitney test. To evaluate the relationship between the percent changes in BMD and laboratory data, we used the partial Spearman correlation coefficient after adjusting for age and the follow-up period. The differences in the percent changes in BMD before and after treatment for both groups were expressed as means, adjusted for age and the follow-up period by using analysis of covariance. All statistical analyses were performed using PASW Statistics 20.0 (SPSS Inc., Chicago, IL). All analyses were 2-sided, and *P* <.05 were considered statistically significant.

## 3 Results

Of the 49 patients, 34 underwent gastrectomy and 15 underwent endoscopic treatment. The clinical characteristics of the study patients according to treatment modality are presented in Table 1. The mean age of the patients tended to be higher in the endoscopic treatment group than in the gastrectomy group (*P* = .057). There were no differences between the 2 groups with regard to BMI, smoking and alcohol habits, chemotherapy, and comorbid disease status. In addition, baseline BMD and biochemical data did not differ between the gastrectomy and endoscopic treatment groups. The mean and median follow-up intervals for measurement of BMD among the study patients were 32.6 months (SD, 16.5) and 31.0 months (interquartile range, 21.5, 41.0), respectively, and these values did not differ between the 2 groups. The follow-up BMDs of the femoral neck and total hip were lower in the gastrectomy group than in the endoscopic treatment group (*P* = .010 and .011, respectively). In addition, the follow-up BMD of the lumbar spine tended to be lower in the gastrectomy group than in the endoscopic treatment group (*P* = .054). Among the follow-up biochemical data, the serum phosphorus level was higher in the gastrectomy group than in the endoscopic treatment group (*P* = .017).

**Table 1:**
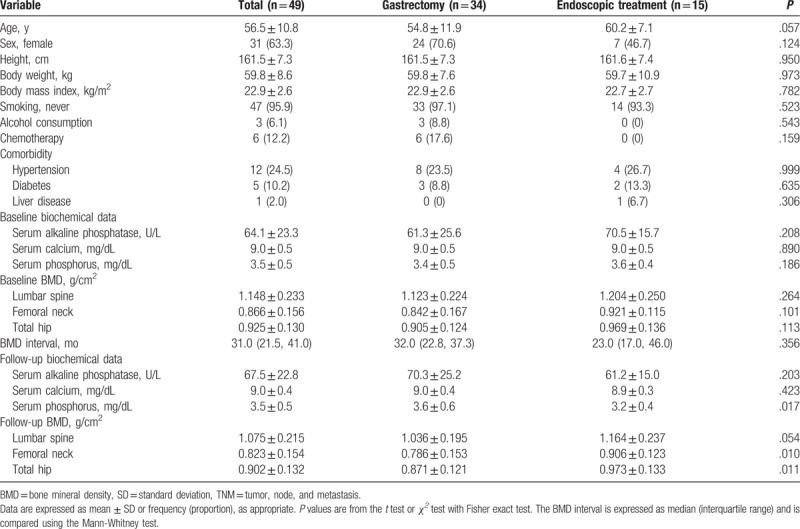
Clinical characteristics of the patients with gastric cancer (TNM stage 1) according to the treatment modality.

The relationships between the percentage changes in the baseline and follow-up BMD measurements and biochemical data are presented in Table 2. In the gastrectomy group, the change in BMD of the lumbar spine was negatively correlated with the change in the serum alkaline phosphatase level and the change in BMD of the total hip was negatively correlated with the change in the serum calcium level. However, in the endoscopic treatment group, no correlation was noted between BMD and biochemical data.

**Table 2:**
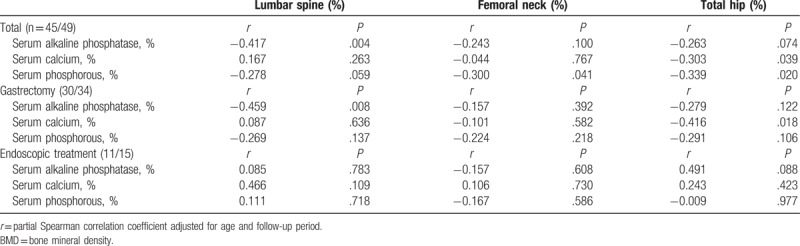
Relationship between the percentage changes in BMD and the serum alkaline phosphatase, calcium, or phosphorous level.

**Figure 1:**
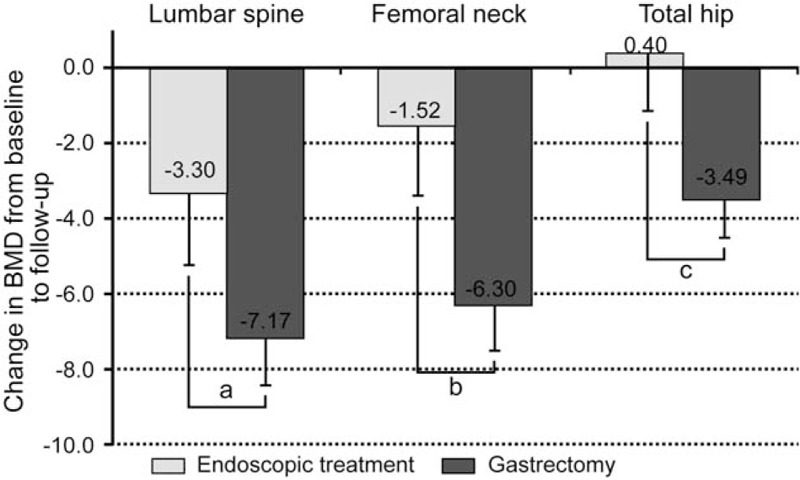
Percentage changes in BMD from baseline to follow-up in the endoscopic treatment group (n = 15) and gastrectomy group (n = 34). BMD values are calculated as mean ± SE and are adjusted for age and follow-up period. ^a^*P* = .028, ^b^*P* = .022, and ^c^*P* = .137 between the 2 groups. BMD = bone mineral density, SE = standard error.

The percentage changes in BMD from baseline to follow-up which adjusted for age and follow-up period are presented in Figure 1. In the endoscopic treatment group, the percentage changes in BMD for the lumbar spine, femoral neck, and total hip were −3.30%, –1.52%, and 0.40%, respectively. In the gastrectomy group, the percentage changes in BMD for the lumbar spine, femoral neck, and total hip were −7.17%, –0.30%, and −3.49%, respectively, with all BMD values showing decreases. The extents of bone loss of the lumbar spine and femoral neck were greater in the gastrectomy group than in the endoscopic treatment group (*P* = .028 and .022, respectively); however, the extent of bone loss of the total hip did not differ between the gastrectomy and endoscopic treatment groups.

## 4 Discussion

The current study found that the magnitude of bone loss was greater in the gastrectomy group than in the endoscopic treatment group.

The etiology of postgastrectomy bone disease still remains unclear. Malabsorption and a decrease in the dietary intake of vitamin D and calcium are major causes of bone loss. Poor absorption of vitamin D and calcium results in secondary hyperparathyroidism, which increases the rate of bone loss and leads to osteomalacia or osteoporosis. Postgastrectomy bone disorders include osteomalacia, osteoporosis, or a combination of both.^[8,9]^ A German study reported an overall rate of vertebral fractures or osteopenia of as high as 55% after gastrectomy.^[1]^ In addition, a Korean study reported a high rate of osteoporosis and vertebral bone deformity among patients with gastric cancer after gastrectomy, regardless of the interval after gastrectomy and operation type, and reported that the prevalence of osteoporosis in the lumbar spine was 38.3% and in the femoral neck was 15.0%.^[10]^

In the current study, we found a decrease in BMD among early stage gastric cancer survivors after treatment, which was greater in the gastrectomy group than in the endoscopic treatment group. In the gastrectomy group, the percentage changes in BMD from baseline to follow-up were −7.17% in the lumbar spine, −6.30% in the femoral neck, and −3.49% in the total hip. Decreases in trabecular and cortical bone densities have been reported to be 2% to 4% and 1% to 2% per year, respectively, during the rapid phase of menopausal bone loss, and age-related bone loss has been reported to slow to a rate of 0.5% to 0.75% per year.^[11]^ In the current study, considerable changes in BMD were noted in the gastrectomy group, whereas the changes in BMD in the endoscopic treatment group might be similar to those in the general population.

Our results are consistent with those of a previous Korean study that investigated 36 gastric cancer survivors who underwent DXA evaluation before and 1 year after gastrectomy.^[5]^ They reported that the percentage changes in BMD of the lumbar spine, total hip, femoral neck, and trochanter from baseline to 1 year later were −5.7%,5.4%, –6.6%, and –8.7%, respectively, which were greater than those identified in the currentstudy with a 3-year BMD follow-up interval. These authors enrolled patients with cancers at various stages (stages I, II, and III), and 39% of the study patients received chemotherapy. Therefore, cancer burden and cancer-related treatments might have further decreased the BMD.

Contrary to the results of our study, a longitudinal study conducted in Sweden with 22 gastric cancer survivors after total gastrectomy reported that there was no difference in BMD between those who underwent gastrectomy and a reference population at 5 and 8 years after gastrectomy.^[4]^ This difference may have occurred because postoperatively, all patients in the Swedish study were educated and followed by a trained dietitian and the Swedish population has a high intake of dairy products, which are fortified with vitamin D.

It has been reported that the consumption of dairy products that are high in calcium and vitamin D is lower in Asian countries than in Western countries.^[12]^ According to the Korea National Health and Nutrition Examination Survey VI (2013), the mean dietary intake of calcium among the Korean population was only 70% of the recommended dietary allowance per day. In addition, vitamin D insufficiency is a very common problem in Korea, and the prevalence of vitamin D insufficiency, defined as a serum 25-hydroxyvitamin D [25(OH)D] level of less than 20 ng/mL, is reported to be 47.3% in men and 64.5% in women.^[13]^ This suggests that the Korean population might be more susceptible to a decrease in BMD after gastrectomy than the Western population.

In the current study, the serum alkaline phosphatase level increased after gastrectomy and the change in the BMD of the lumbar spine was negatively correlated with the change in the serum alkaline phosphatase level. The serum alkaline phosphatase level has been previously used as a marker for bone formation and to detect postgastrectomy osteomalacia.^[14]^ However, the relationship between the serum alkaline phosphatase level and osteomalacia is still controversial. Several studies have reported that the serum alkaline phosphatase level increased after partial and total gastrectomy,^[15,16]^ whereas other studies reported that the serum alkaline phosphatase level was within the normal range after gastrectomy.^[3,17]^ Further studies are needed to assess the usefulness of laboratory tests of bone mineral metabolism to diagnose postgastrectomy bone disease.

Our study had several strengths. First, we demonstrated the differences in BMD after treatment for gastric cancer, according to the treatment modality (gastrectomy vs endoscopic treatment). Second, we focused on early stage gastric cancer survivors to avoid the effects of cancer burden and chemotherapy toxicity on bone metabolism. Moreover, we adjusted age and the postoperative follow-up period. Despite these strengths, the study had some limitations. First, the body weights after treatment and body composition data of the study patients were not available; thus, we could not adjust for the changes in body weight before and after treatment. After gastrectomy, loss of body weight that consist of lean body mass and fat mass commonly occurred because of poor nutritional intake and malabsorption,^[18]^ whereas endoscopic treatment could maintain body weight.^[19]^ Both lean body mass and fat mass are known to influence BMD by mechanical force and hormonal factors.^[20]^ Therefore, weight loss after gastrectomy could be an explanatory factor that greater decrease in BMD compared to endoscopic treatment. However, most weight loss developed during immediate postoperative period, and stabilized overtime. Recent long-term retrospective study revealed that the percent weight loss which was the difference between the baseline and postoperative 1-year body weight, divided by the baseline body weight is not associated with fracture after gastrectomy among gastric cancer patients.^[21]^ Further studies are necessary to clarify the influence of weight loss after gastrectomy on bone health. Second, we did not measure the dietary intake of calcium and vitamin D, and the serum 25(OH)D level, which might influence bone mineral metabolism. Although we excluded the subjects with the treatment for osteoporosis, prior history of fracture, or hormone replacement therapy, and the diseases that would affect BMD such as renal failure, thyroid disease, liver cirrhotic disease, and immobility, there was no available data that about rheumatoid arthritis or use of oral corticosteroids that were known risk factors of osteoporosis. Third, this study had a retrospective study design, and the subjects could not be randomly assigned to both treatment groups. Therefore, we could not exclude selection bias or unknown confounders that might affect to change of bone density in both groups.

In the current study, we confirmed that BMD is lower after gastrectomy than after endoscopic treatment among early stage gastric cancer survivors. The magnitude of bone loss was greater in the gastrectomy group than in the endoscopic treatment group. Therefore, endoscopic treatment might have an advantage regarding maintenance of bone health among gastric cancer patients. In addition, physicians should keep a watch for decrease in BMD and consider screening for osteoporosis among gastric cancer survivors.
